# Patient radiation doses in an angiography suite at a tertiary hospital in Pretoria

**DOI:** 10.4102/jcmsa.v4i1.279

**Published:** 2026-04-21

**Authors:** Mmatlou D. Seema, Alireza Dehghan-Dehnavi, Hanyani Lowane

**Affiliations:** 1 Department of Diagnostic Radiology and Imaging, School of Medicine, Sefako Makgatho Health Sciences University, Pretoria, South Africa

**Keywords:** diagnostic reference level, kerma air product, fluoroscopy time, interventional radiology, interventional neuroradiology, radiation dose optimisation

## Abstract

**Background:**

The rising demand for interventional radiology procedures (IRPs) performed under fluoroscopic guidance increases the risk of harmful radiation effects for patients and staff. Radiation dose optimisation using diagnostic reference levels (DRLs) is necessary to keep radiation ‘as low as reasonably achievable (ALARA)’. Establishment of DRLs is required for radiation imaging centres; however, only a few have published them in South Africa. This study aimed to establish local diagnostic reference levels (LDRLs) for IRPs and compare them with DRLs of local and international imaging centres.

**Methods:**

A retrospective descriptive study was conducted at a single imaging centre. The IRPs performed on adults between 01 January 2023 and 31 December 2023 were considered. Ethical clearance was obtained before data collection. Data on fluoroscopy time (FT) and kerma air product (KAP) were obtained from a procedure register book. The third quartile (75th percentile) of the dataset for KAP and FT of every IRP indicated the LDRLs.

**Results:**

A total of 375 IRPs were analysed. The most frequently performed IRP was cerebral interventions (21%, *n* = 80). Aortic interventions recorded the highest dose LDRL (KAP = 252.5 Gy/cm^2^), followed by cerebral interventions (KAP = 211.3 Gy/cm^2^). Cerebral interventions recorded the longest FT (56.8 min), followed by aortic interventions (36.2 min), both of which exceeded the local centres’ DRL ranges. Permanent catheterisation (KAP = 10.7 Gy/cm^2^) also exceeded the international DRL range.

**Conclusion:**

Established LDRLs for IRPs were mostly within local and international DRL ranges.

**Contribution:**

The study contributed to the establishment of local, national and regional DRLs, which assisted imaging centres in optimising radiation doses for IRPs.

## Introduction

The number of interventional radiology procedures (IRPs) performed under fluoroscopic guidance is increasing globally.^1^ This is mainly because their execution is less invasive than traditional open surgical techniques. Other advantages of IRPs include shorter recovery periods and reduced hospital stays compared to surgical procedures.^2^ The IRPs are typically performed in an angiography suite, an interventional theatre fitted with a fixed X-ray fluoroscopy machine that guides diagnostic and interventional procedures.^3,4^ The advantage of fluoroscopy over other imaging modalities is its ability to acquire real-time images with a high temporal resolution.^5^

The IRPs performed by radiologists in the angiography suite can be categorised as interventional neuroradiology (INR) and body interventional radiology (IR). The INR procedures include diagnostic cerebral angiography, cerebral aneurysm, arteriovenous fistula and arteriovenous malformation embolisation.^6^ The IR procedures can be further divided into vascular and non-vascular procedures. Vascular procedures are vessel angioplasty, stenting and solid organ embolisation (i.e., spleen, liver or kidney). Non-vascular body IR procedures encompass percutaneous transhepatic cholangiography, biliary stenting, percutaneous transhepatic biliary drainage (PTBD), ureteric stenting, nephrostomy and pigtail catheter drainage for various conditions.^7^

Fluoroscopy-guided IR procedures may take hours to complete, depending on the complexity of the case.^6^ Because of the need for cine acquisition and prolonged screening, fluoroscopic assessments may inadvertently expose patients to elevated radiation doses, thereby augmenting the risk of eliciting tissue reactions (formerly known as deterministic effects).^1,5^ Examples of tissue reactions are skin injuries (i.e., erythema, ulcer or necrosis)^8^ and eye lens injuries (i.e., cataract) in patients who underwent head IRPs.^9^ Interventionalists also face a risk of developing cataracts in the eye.^10^ While tissue reactions occur above a certain radiation dose threshold, stochastic effects may be triggered by any amount of radiation exposure (no threshold).^1,11^ They include leukaemia (except chronic lymphocytic leukaemia) and radiation-related malignancies (i.e., carcinoma of the thyroid, breast, lung, bowel, bone and skin).^12,13^ Consequently, emphasis must be placed on IRPs to minimise radiation dose to the patients.^14^ Effective radiation management is achieved through optimisation, justification and dose limitation while maintaining the integrity and quality of the image.^15^

The utilisation of diagnostic reference levels (DRLs) for IRPs optimisation purposes was conceptualised by the International Commission on Radiological Protection (ICRP).^15^ Diagnostic reference levels are notional values specific to a particular investigation that can help identify exposures that are extraordinarily low or high.^15^ According to ICRP publication 135, the median of the distribution of DRL values for a specific procedure at a facility is termed the typical value. These values are used when a centre is too small to establish local diagnostic reference levels (LDRLs).^15^ Local diagnostic reference levels are the third quartile (75th percentile) of median values for X-ray rooms at a facility or a few facilities in a local area.^15^ The national diagnostic reference levels (NDRLs) are the third quartile of median values for a representative sample of X-ray facilities across an entire country, whereas the regional DRLs pertain to a representative sample of the NDRLs of several countries in a geographical region.^15^

The necessary radiation dose parameters for determining DRL are fluoroscopy time (FT), reference air kerma (K_a,r_) and dose area product (DAP) or kerma area product (KAP).^5,16^ These parameters are automatically recorded by the fluoroscopy unit after each procedure.^1,5^ Local diagnostic reference level is indicated by the third quartile (75th percentile) of the dataset for the K_a,r_, KAP and FT of every fluoroscopically guided IRP. The DRL values should not be exceeded in the context of prudent, routine practice and can be applied at the local, national or regional levels.^1^

The Directorate of Radiation Control (DRC) instituted DRLs as a legal obligation in 2015 and required institutions to develop centre-wide LDRLs for specific procedures.^14^ Subsequently, X-ray machine licensees were mandated to appoint a physicist (medical) to develop and enforce a programme to optimise DRLs for all fluoroscopy-guided IRPs.^3,14^ The DRC regulations state that LDRLs are also necessary to establish NDRLs. Despite these requirements, only a few centres have established and published LDRLs for IRPs.^1^ The absence of DRLs may inadvertently lead to high radiation exposure for patients with no recourse.^5^

The available DRL data predominantly originate from high-income countries according to the World Bank criteria.^16^ However, these data do not adequately represent low-income and lower-middle-income countries, where over 80% of the global population resides. The World Bank classified South Africa as a lower-middle-income country.^16^ Published literature on DRLs in sub-Saharan Africa is markedly limited when compared with that in high-income countries. In 2015, fewer than 21% of the 135 lower-middle-income countries published DRLs for fluoroscopy-guided procedures.^1,16^

The NDRLs are yet to be established in South Africa. Their establishment depends on the comprehensive development of LDRLs of individual centres throughout all nine provinces.^1,14^ A review conducted in 2021 regarding South Africa’s DRL data revealed that only three (Gauteng, Free State, and Western Cape) out of the nine provinces published any DRL information.^14^ Thus, comprehensive data on LDRLs representing all nine provinces of South Africa are mandatory for successfully developing NDRLs. A few publications have established LDRLs for IRPs within South Africa.^16^ A study from Tygerberg Hospital in Stellenbosch (Western Cape province) by Malan et al. in 2020 determined the LDRL for prevalent IRPs in South Africa and compared it with internationally published data.^17^ They reported that LDRLs for all IR procedures did not exceed published levels.^17^

The most recent similar investigation from Soweto (Gauteng province), South Africa, was conducted at Chris Hani Baragwanath Academic Hospital (CHBAH) and published in 2023 by Slave et al.^1^ It sought to develop LDRLs and compared them with other published DRLs. The results showed that LRDLs for uterine artery embolisation (1463.8 Gy/cm^2^), diagnostic cerebral angiograms (209.3 Gy/cm^2^) and interventional cerebral angiograms (275 Gy/cm^2^) at CHBAH exceeded the international and local DRLs.^1^ The published DRL ranges were as follows: uterine artery embolisation (118.4 Gy/cm^2^ – 214 Gy/cm^2^), diagnostic cerebral angiogram (55 Gy/cm^2^ – 87.5 Gy/cm^2^), interventional cerebral angiogram (63 Gy/cm^2^ – 233.4 Gy/cm^2^), bronchial artery embolisation (73 Gy/cm^2^ – 131.4 Gy/cm^2^), PTBD (23 Gy/cm^2^ – 145 Gy/cm^2^) and unilateral nephrostomy (10 Gy/cm^2^ – 47 Gy/cm^2^).^1,17^

There are no prior calculations or publications of LDRLs for IRPs in the IR angiography suite at Dr. George Mukhari Academic Hospital (DGMAH), rendering the hospital non-compliant. Hence, this study aimed to establish the DRLs for IRPs at DGMAH to comply with the statutory requirements and guidelines, contribute to the establishment of NDRL and reduce adverse radiation effects from overexposure.

## Research methods and design

A retrospective, quantitative, descriptive, cross-sectional study was conducted at DGMAH. This public academic hospital is located in Pretoria (South Africa) and is affiliated with the Sefako Makgatho Health Sciences University. The hospital has a total of 1652 beds. A convenience sampling technique was employed, in which consecutive IRPs performed between 01 January 2023 and 31 December 2023 were included. Achieved radiation doses of IRPs performed in the angiography suite on patients above the age of 18 years were included. The procedures encompassed both diagnostic and therapeutic interventions involving INR and body IR. Body IRPs included non-vascular and vascular fluoroscopy-guided interventions.

The IRPs performed in an angiography theatre without using fluoroscopic guidance were excluded from the study. Patients under 18 years of age were excluded. Register entries lacking KAP values were not considered. Equally, illegible dose entries were excluded. The IRPs with fewer than 15 cases were excluded. Although ICRP recommends at least 20 examinations for DRL estimation, it also encourages flexibility when fewer procedure data are available.^15^ Several recent local publications used a 15 IRP threshold for establishing DRLs; hence, the adoption of this threshold.^1,5,17^ This also enables appropriate comparison of LDRL values among local facilities.

### Data collection

The relevant radiation dose parameters (FT and KAP) required for DRLs calculation were collected from the procedure register book. These parameters were systematically recorded by the fluoroscopy machine after completion of every IRP.^1^ Thereafter, the doses were manually entered by radiographers into the procedure register book. The DRC mandates that both KAP and FT must be recorded.^5^ Thus, radiographers did not capture K_a,r_ into the register book, and it was not collected.

### Quality assurance and dosimetry

The fluoroscopy unit utilised for IRPs during the period under review was a biplane Phillips Allura Xper (FD20/15), commissioned in 2015. The machine had built-in K_a,r_ and KAP at a specific point on the beam (X-ray) and automatically computed the radiation dose for each X-ray tube, along with FT.^5^ Subsequently, the fluoroscopy machine produced a radiation structured dose report (RSDR) for dosimetric parameters (i.e., KAP, K_a,r_ and FT) pertinent to each procedure undertaken.^1,5^

The KAP represents the cumulative measure of air kerma across the entirety of the X-ray beam emitted from the X-ray tube.^2^ Therefore, it provides an indirect measure of radiation exposure, expressed in units of grey per square centimetre (mGy.cm^2^ or Gy.cm^2^).^1,2^ Kerma is an acronym for kinetic energy transferred per unit mass. It refers to the energy imparted from an X-ray beam per unit mass of air within a designated irradiated air volume, and it quantifies the radiation dose administered to that air volume.^1,2^

Air kerma (K_a,r_) signifies the air kerma accumulated at the interventional reference point in relation to the X-ray tube focal spot. The K_a,r_ is also known as the cumulative dose or reference dose, and its unit is grey (Gy).^1,2^ The reference point is situated on the central axis, 15 cm from the system isocentre, along the trajectory of the X-ray point of focus.^5^ Fluoroscopy time encompasses the total duration spent utilising fluoroscopy.^5^ Rigorous quality control assessments are required annually to ensure the objectivity, reliability and validity of the parameter values produced. The author verified by inspecting filed reports that quality assurance measures were consistently maintained throughout the study for each IRP.^5^

The mean deviation in the calibration of the DAP metre, the mean deviation of the dose rate at the entrance surface of the phantom, and the mean deviation regarding the precision of the FT were all in accordance with the guidelines and manufacturers’ specifications.^18^ Dosimetric values were considered valid and reliable as the quality control outcomes remained within acceptable tolerance parameters throughout the study period.

### Data analysis

**FIGURE 1:**
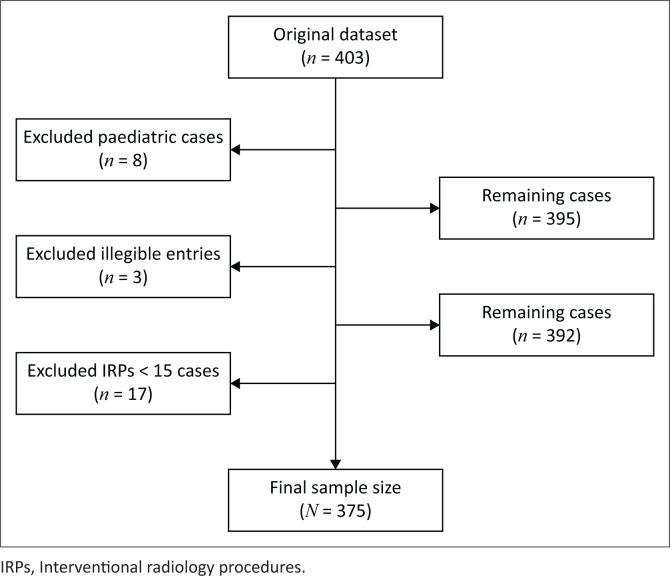
Data cleaning from the original dataset to the final sample size.

A total of 403 IRPs were conducted during the investigation period, and 93% (*n* = 375) met the inclusion criteria, whereas 7% (*n* = 28) were excluded, as depicted in Figure 1. Excluded data were paediatric cases (2%, *n* = 8), illegible entries (1%, *n* = 3) and IRPs with less than 15 cases (4%, *n* = 17). The distribution of the included data is demonstrated in Figure 3.

The collected data were entered into a spreadsheet (Microsoft Excel) for analysis. Previous studies have successfully established DRLs using Microsoft Excel.^1,17^ Hence, a similar analytical approach was adopted in this study.

The data were cleaned by identifying and correcting errors (i.e. duplicates) in the dataset. Verification of the minimum and maximum values for each variable was also conducted before analysing the data. The Microsoft Excel frequency function was utilised to organise the distribution of IRPs. Statistical analysis (descriptive) was undertaken to measure the 25th percentile, 50th percentile (median) and 75th percentile of every dosimetric variable.^1,17^ The LDRL was indicated by the third quartile (75th percentile) of the dataset for FT and KAP for each fluoroscopy-guided procedure.^15^ The LDRLs were subsequently compared with established international and other local DRLs.^1,17^

### Ethical considerations

Ethical clearance was obtained from the Sefako Makgatho University Research Ethics Committee (SMUREC) before the commencement of data collection (SMUREC/M/79/2025:PG). Permission for data collection from the hospital records was granted by the Head of Department and Clinical Executive Director of DGMAH. Patient personal information and identifiers were not collected. All data collected for this study was securely stored on a password-protected computer and will be retained for 2 years. After that, it will be discarded.

## Results

### Frequency distribution of interventional radiology procedures

**FIGURE 2:**
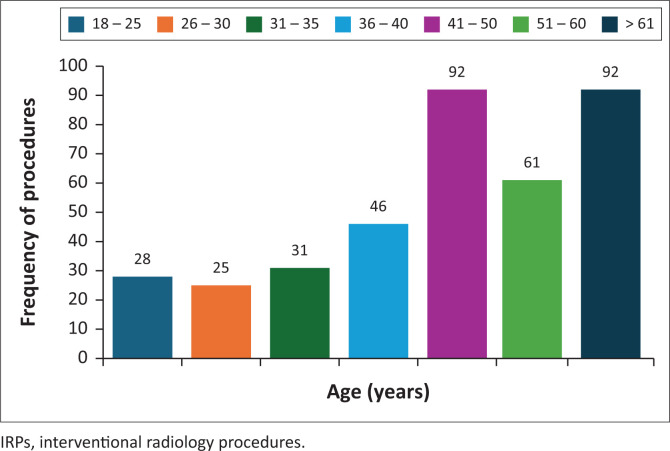
Frequency of IRPs according to age groups.

Most IRPs were performed on male patients (54%, *n* = 201), whereas female patients accounted for 46% (*n* = 174). A quarter (*n* = 92) of IRPs were performed in the age groups 41–50 years and above 61 years, respectively, and together accounted for approximately 50% of the total IRPs. The younger age groups (typically healthy) had fewer IRPs, with the age group 26–30 years having the lowest share at 7% (*n* = 25). The overall trend shows that more IRPs were performed in older age groups (prone to chronic illness), with a steep increase beginning at age 40 years old, as shown in Figure 2.

**FIGURE 3:**
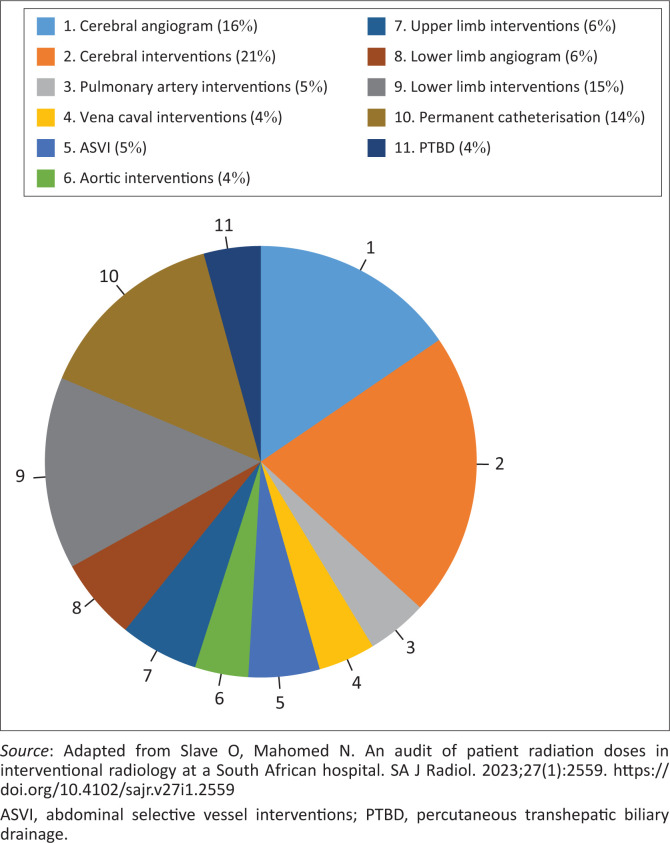
Frequency distribution of interventional radiology procedures performed during the period under review.

The most frequently performed IRPs were cerebral interventions, which accounted for 21% (*n* = 80) of the total, as depicted in Figure 3. Cerebral angiograms followed it at 21% (*n* = 58). The least performed IRPs included in the study were PTBD at 4% (*n* = 16), vena caval interventions at 4% (*n* = 16) and aortic interventions also at 4% (*n* = 15). The excluded IRPs with fewer than 15 cases comprised nephrostomies, ureteric stent insertions, upper limb angiograms and extracranial head and neck interventions.

### Dosimetric parameters

Kerma air product and FT measurements required for establishing DRLs of each IRP are presented in Table 1. Local diagnostic reference level for each IRP was recorded in the 75th percentile column, indicating relatively high DRLs for aortic and cerebral interventions.

**TABLE 1:** Radiation dose parameter for this study.

Iterventional procedure	Total IRPs (*n*)	Dose area product (GY/cm^2^)					Filmorescopy time (min)				
		25th percentile	50th percentile	75th percentile	IQR	75th: 50th	25th percentile	50th percentile	75th percentile	IQR	75th: 50th
Cerebral angiogram	58	46.1	62.4	81.1	35	1.3	5.3	8.8	14.0	8,7	1.6
Cerebral interventions	80	112.0	142.8	211.3	99,3	1.5	26.4	42.1	56.8	30,3	1.4
Pulmonary artery interventions	17	28.3	43.1	55.9	27,6	1.3	11.9	15.3	20.5	8,6	1.3
Vena caval interventions	16	12.3	39.8	70.9	58,5	1.8	3.2	6.2	9.4	6,2	1.5
ASVI	20	36.0	107.6	139.2	103,2	1.3	9.2	14.7	34.1	24,9	2.3
Aortic interventions	15	124.4	244.3	252.5	128	1.0	12.8	25.5	36.2	23,5	1.4
Upper limb interventions	22	3.9	8.6	17.1	13,1	2.0	1.4	4.3	9.3	7,9	2.2
Lower limb angiogram	23	3.0	8.6	18.8	15,8	2.2	1.1	42.0	7.2	6,1	1.7
Lower limb interventions	54	13.7	23.4	58.1	44,4	2.5	6.2	14.8	21.5	15,3	1.5
Permanent catheterisation	54	25.0	42.0	10.7	8,2	25.0	0.4	13.0	3.3	2,8	2.6
PTBD	16	5,3	6.4	8.3	3,1	1.3	1.5	2.3	3.5	2	1.5

*Source*: Adapted from Slave O, Mahomed N. An audit of patient radiation doses in interventional radiology at a South African hospital. SA J Radiol. 2023;27(1):2559. https://doi.org/10.4102/sajr.v27i1.2559

IQR, interquartile range; IRPs, interventional radiology procedures; ASVI, abdominal selective vessel interventions; PTBD, percutaneous transhepatic biliary drainage.

Aortic interventions recorded the highest dose LDRL (KAP = 252.5 Gy/cm^2^), followed by cerebral interventions (KAP = 211.3 Gy/cm^2^), as shown in Table 1. Cerebral interventions recorded the longest FT (56.8 min), followed by aortic interventions (36.2 min) and abdominal selective vessel interventions (ASVI) (34.1 min). The lowest LDRL dose was recorded for PTBD (KAP = 8.3 Gy/cm^2^), preceded by permanent catheterisation (KAP = 10.7 Gy/cm^2^). Permanent catheterisation interventions recorded the shortest FT LRDL (3.3 min), preceded by PTBD (3.5 min). Cerebral interventions LDRLs (56.8 min) for FT were the longest of all IRPs. Cerebral interventions and ASVI (34.1 min) exceeded the published DRL ranges of other institutions in South Africa (25–34.1 min and 28.3–29 min, respectively).^1,17^

Variation with a dose parameter was indicated by the 75th:50th centile ratio. Aortic interventions had the narrowest KAP dose variation (1.0) for each parameter, followed by cerebral angiogram (1.3), together with pulmonary artery interventions (1.3), ASVI (1.3) and PTBD (1.3). Lower limb interventions (2.5) and permanent catheterisation (2.5) recorded the widest KAP dose variation, followed by lower limb angiogram (2.2) and upper limb interventions (2). A wide parameter variation was observed in procedures commonly performed by trainees (registrars and fellows). These results are consistent with findings from similar studies, which demonstrated a wide variation in dose parameters because of different procedure approaches employed by inexperienced operators.^1,17^

## Discussion

The established KAP LDRL at DGMAH were within the published international DRL ranges, except for permanent catheterisation and upper limb interventions. Permanent catheterisation KAP LDRL (10.7 Gy/cm^2^) was above the international range (0.9 Gy/cm^2^ – 2.9 Gy/cm^2^). Upper limb interventions KAP LDRL (17.1 Gy/cm^2^) were also higher than the international range (10 Gy/cm^2^ – 14.2 Gy/cm^2^). The two procedures were typically reserved for trainees who were inexperienced in performing IRPs. These findings are consistent with published DRL studies, which established that procedures performed by inexperienced operators were associated with higher radiation doses.^1,17^

The highest international cerebral intervention LDRL (104 min) for FT was recorded in a study done in India.^19^ The discrepancies in DRL values across nations and regions may be attributed to differences in imaging equipment, local procedure protocols and operator techniques.^15^ Cerebral interventions DRLs were ranked high among all IRPs for dose (KAP) and radiation exposure duration (FT) in this research, consistent with several other publications.^1,17,19,20,21,22,23,24,25^ This is presumably associated with the complexities of cerebral interventions. Other studies that published DRLs did not include data for cerebral interventions, as shown in Table 2 and Table 3.^2,26,27,28,29,30,31,32,33,34^

**TABLE 2:** LDRLs for KAP at DGMAH compared with published data.

Interventional procedure	This study	DRL Range SA	Slave et al.^1^	Malan et al.^17^	DRL Range Int.	Issahaku et al.^26^	Korir et al.^2^	Arabi et al.^27^	Rizk et al.^20^	Varghese et al.^21^	Rana et al.^19^
Country	South Africa	South Africa	South Africa	South Africa	International	Ghana	Kenya	Saudi Arabia	Lebanon	India	India
Cerebral angiogram	81.1	55-209.3	209.3	55	41-978	111.8	978	-	71	100.3	122
Cerebral interventions	211.3	63-275	275	63	152.9-368.3	-	-	-	197	161.7	368.3
Pulmonary artery interventions	55.9	N/A	-	-	N/A	-	-	-	-	-	-
Vena caval interventions	70.9	N/A	-	-	22.3-189	-	189	29.5	55	54.6	-
ASVI	139.2	170-777	777	170	107-571	150.4	-	-	571	154.6	1975
Aortic interventions	252.5	N/A	-	-	198.1-514	198.1	-	-	342	-	-
Upper limb interventions	17.1	17.1-30	-	30	10-142	-	-	14.2	-	-	-
Lower limb angiogram	18.8	18.8-46.5	-	46.5	3-398	-	398		47	-	20.4
Lower limb interventions	58.1	58.1-73	-	73	14.1-119	-	-	36.3	83	52.3	48.1
Permanent catheterisation	10.7	N/A	-	-	0.9-2.9	-	-	2.7	-	-	-
PTBD	8.31	24-46	24	46	3.1-145	-	117	79.3	145	23	-

*Source*: Adapted from Slave O, Mahomed N. An audit of patient radiation doses in interventional radiology at a South African hospital. SA J Radiol. 2023;27(1):2559. https://doi.org/10.4102/sajr.v27i1.2559

Note: Also see the full reference list of Seema MD, Dehghan- Dehnavi A, Lowane H. Patient radiation doses in an angiography suite at a tertiary hospital in Pretoria. J Coll Med S Afr. 2026;4(1), a279. https://doi.org/10.4102/jcmsa.v4i1.279

DRL, diagnostic reference levels; SA, South Africa; Int, international; N/A, not applicable; ASVI, abdominal selective vessel interventions; PTBD, percutaneous transhepatic biliary drainage.

**TABLE 3:** Dr. George Mukhari Academic Hospital local diagnostic reference levels for kerma air product compared with published data.

Interventional procedure	Pimenta et al.^28^	Jones et al.^29^	Tristram et al.^22^	Papanastassion et al.^30^	Schegerer et al.^31^	Schegerer et al.^23^	Etard et al.^24^	Heilmaier et al.^32^	Ruiz-Cruces et al.^33^	Erskine et al.^25^	Zotova et al.^34^
Country	Portugal	USA	Germany	Greece	Europe	Germany	France	Switzerland	Spain	Australia	Bulgaria
Cerebral angiogram	-	-	100.6	70.2	-	-	87.5	-	-	106.3	41
Cerebral interventions	-	-	186.8	-	-	192	233.5	-	-	152.9	-
Pulmonary artery interventions	-	-	-	-	-	-	-	-	-	-	-
Vena caval interventions	-	31.7	-	-	-	-	-	22.3	-	27.7	23
ASVI	331.6	299.8	2228	-	-	-	322.6	314.1	107	168.8	290
Aortic interventions	-	-	-	-	-	208	-	237.8	-	514	-
Upper limb interventions	-	-	-	-	-	-	-	-	-	10	-
Lower limb angiogram	-	-	40.7	-	-	-	72	3	78	75.1	47
Lower limb interventions	19.3	-	-	-	26	27.5	-	14.1	119	15.8	42
Permanent catheterisation	-	-	1.4	-	-	-	1.5	2.9	-	0.9	-
PTBD	20.2	66	3.1	53.8	23	-	33.5	60.5	30	-	42

*Source*: Adapted from Slave O, Mahomed N. An audit of patient radiation doses in interventional radiology at a South African hospital. SA J Radiol. 2023;27(1):2559. https://doi.org/10.4102/sajr.v27i1.2559

Note: Also see full reference list of Seema MD, Dehghan- Dehnavi A, Lowane H. Patient radiation doses in an angiography suite at a tertiary hospital in Pretoria. J Coll Med S Afr. 2026;4(1), a279. https://doi.org/10.4102/jcmsa.v4i1.279

DGMAH, Dr. George Mukhari Academic Hospital; KAP, kerma air product; LDRL, local diagnostic reference levels; USA, United States of America; ASVI, abdominal selective vessel interventions; PTBD, percutaneous transhepatic biliary drainage.

Table 2 and Table 3 demonstrate that the established LDRLs for KAP were comparable with the published DRLs from other institutions in South Africa, Africa, the Middle East, Asia, Europe and the United States of America. A similar trend was demonstrated for FT LDRLs in Table 4 and Table 5.

**TABLE 4:** Dr. George Mukhari Academic Hospital local diagnostic reference levels for fluoroscopy time compared with published data.

Interventional procedure	This study	DRL Range SA	Slave et al.^1^	Malan et al.^17^	DRL Range Int.	Issahaku et al.^26^	Korir et al.^2^	Arabi et al.^27^	Rizk et al.^20^	Varghese et al.^21^	Rana et al.^19^
Country	South Africa	South Africa	South Africa	South Africa	International	Ghana	Kenya	Saudi Arabia	Lebanon	India	India
Cerebral angiogram	14	14-28.4	28.4	14	8-642	20.5	104		8	23.9	64.2
Cerebral interventions	56.8	25-34.1	34.1	25	28-104	-	-	-	28	83.4	104
Pulmonary artery interventions	20.5	N/A	-	-	N/A	-	-	-	-	-	-
Vena caval interventions	9.4	N/A	-	-	2.8-31.9		5	6	6	31.9	-
ASVI	34.1	283-29	28.3	29	3-57	20.5	-	-	33	52.9	57
Aortic interventions	36.2	N/A	-	13	23-34.7	23	-		23	-	-
Upper limb interventions	93	93-13	-	7	115-17	-	-	17	-	-	-
Lower limb angiogram	7.2	7-7.2	-	13	4-48	-	48	-	4	-	-
Lower limb interventions	21.5	13-21.5	-	-	13-78.6	-	-	30.4	16	10.6	13
Permanent catheterisation	3.3	N/A	-	-	0.4-1.7	-	-	1.7	-	-	78.6
PTBD	35	62-20	6.2	20	1-69	-	69	20.9	20	20.6	-

*Source*: Adapted from Slave O, Mahomed N. An audit of patient radiation doses in interventional radiology at a South African hospital. SA J Radiol. 2023;27(1):2559. https://doi.org/10.4102/sajr.v27i1.2559

Note: Also see full reference list of Seema MD, Dehghan- Dehnavi A, Lowane H. Patient radiation doses in an angiography suite at a tertiary hospital in Pretoria. J Coll Med S Afr. 2026;4(1), a279. https://doi.org/10.4102/jcmsa.v4i1.279

DRL, diagnostic reference levels; SA, South Africa; Int, international; N/A, not applicable; ASVI, abdominal selective vessel interventions; PTBD, percutaneous transhepatic biliary drainage.

**TABLE 5:** Dr. George Mukhari Academic Hospital local diagnostic reference levels for fluoroscopy time compared with published data.

Interventional procedure	Pimenta et al.^28^	Jones et al.^29^	Tristram et al.^22^	Papanastassiou et al.^30^	Schegerer et al.^31^	Schegerer et al.^23^	Etard et al.^24^	Heilmaier et al.^32^	Ruiz-Cruces et al.^33^	Erskine et al.^25^	Zotova et al.^34^
Country	Portugal	USA	Germany	Greece	Europe	Germany	France	Switzerland	Spain	Australia	Bulgaria
Cerebral angiogram	-	-	15.3	9.2	-	-	10.3	-	-	7.5	12.2
Cerebral interventions	-	-	70	-	-	54	62.9	-	-	32	
Pulmonary artery interventions	-	-	-	-	-	-	-	-	-	-	-
Vena caval interventions	-	4.3	-	-	-	-	-	92	-	28	3
ASVI	21.2	35.7	28.7	-	-	-	21.6	20.6	5.6	22.8	3
Aortic interventions	-	-	-	-	-	34.7	-	29.1	-	24	-
Upper limb interventions	-	-	-	-	-	-	-	-	-	11.5	-
Lower limb angiogram	-	-	22.3	-	-	-	5.2	2.1	4	35	2.9
Lower limb interventions	18.3	-	-	-	13	24.5	-	12.1	30	10.25	15
Permanent catheterisation	-	-	0.7	-	-	-	0.8	0.4	-	0.8	-
PTBD	7.2	24.5	1	22.9	10	-	15.7	12.6	17.3	-	9.2

*Source*: Adapted from Slave O, Mahomed N. An audit of patient radiation doses in interventional radiology at a South African hospital. SA J Radiol. 2023;27(1):2559. https://doi.org/10.4102/sajr.v27i1.2559

Note: Also see full reference list of Seema MD, Dehghan- Dehnavi A, Lowane H. Patient radiation doses in an angiography suite at a tertiary hospital in Pretoria. J Coll Med S Afr. 2026;4(1), a279. https://doi.org/10.4102/jcmsa.v4i1.279

USA, United States of America; ASVI, abdominal selective vessel interventions; PTBD, percutaneous transhepatic biliary drainage.

The high radiation dose (KAP) values were not always associated with long radiation exposure (FT). This observation aligns with both global^25,30^ and domestic^1,17^ research findings. It underscores that no specific dose parameters measured at the local level can be reliably extrapolated to infer DRLs at the national or regional level.^1,15^ Thus, it is imperative to assess as many variables as feasible when ‘optimising radiation exposure’.^15^ These sentiments are reiterated in the guidelines proposed by the ICRP.^15^

Data on pulmonary intervention procedures lack comparable international and local benchmarks; as such, it is distinctive to this study. These procedures were mostly performed in patients with pulmonary artery thrombosis for thrombolysis using the EkoSonic Endovascular System (EKOS) from Boston Scientific. Development of a detailed institutional protocol is recommended to guide the process of investigating excessive radiation doses, beginning with an assessment of the equipment, followed by a review of procedural protocols and ultimately an evaluation of operator techniques.^15^

### Limitations

Data for patients under 18 years of age were excluded. Relevant but missing data in the register book were K_a,r_, acquisition parameters (i.e., voltage, current, frame rate and field of view), weight and height. This limited further analysis of results. Because of the absence of K_a,r_ data in the reviewed records, its LDRL for DGMAH could not be established for all IRPs. Furthermore, the relationship of K_a,r_ with KAP and FT was not determined. The body mass index (BMI) was not calculated because weight and height were unavailable. Consequently, the relationship between radiation dose and BMI was not established. The lack of acquisition parameters also limited further interpretation of the results. The missing dosimetric data could not be retrieved from the Picture Archiving and Communication Systems (PACS) because of data loss during PACS vendor changes before the data collection period.

The LDRL for pulmonary artery interventions was lacking in the reviewed local and international publications. As a result, the local and international DRL range for pulmonary artery interventions could not be established, and comparison with DGMAH LDRL was impossible. The published LDRL lacked sufficient data to estimate South Africa’s DRL range for IRPs such as vena cava interventions, aortic interventions, permanent catheterisation, upper limb angiograms and interventions. The IRPs with fewer than 15 entries in the procedure record book (nephrostomy, ureteric stent insertion, extracranial head and neck interventions) were excluded; therefore, their DRLs could not be established.

### Recommendations

Future studies should establish DRLs for patients under 18 years of age. Such investigations will require recording and assessing additional parameters (e.g., acquisition parameters, weight, height and procedure complexity) to enable accurate DRL estimation.^1^ Standardised names for IRPs and agreed weight ranges for adults will enable appropriate comparisons of DRLs across institutions, countries and regions.^1,15^ Unlike for KAP and FT, the DRC does not mandate K_a,r_ recording. However, it is highly recommended that radiographers record it in the procedure register book to enable DRL estimation in case of PACS data loss. A well-coordinated PACS vendor handover is necessary to ensure seamless transition of dosimetric and image data to the new vendor’s data storage. A prospective study may be considered to capture all the required data.

More studies are required to estimate DRLs for pulmonary interventions, vena caval interventions, aortic interventions, permanent catheterisation, upper limb angiograms and upper limb interventions. More in-depth analysis and corrective measures must be undertaken for procedures (i.e., aortic interventions, cerebral interventions, permanent catheterisation and upper limb interventions) that exceeded the published DRL range to uphold the ‘as low as reasonably achievable (ALARA)’ principle and prevent harmful radiation effects. Establishing institutional protocol is recommended to guide the investigation of excessive radiation doses.^15^ Regular revision of DRLs must be performed in accordance with DRC regulations and the ICRP recommendations.^3,15^

## Conclusion

This investigation established LDRLs for IRPs at DGMAH, which were predominantly comparable to South Africa’s and international DRL ranges, except for permanent catheterisation and upper limb interventions, which exceeded these benchmarks. Cerebral interventions and ASVI exceeded the local DRL ranges but remained within international ranges. These procedures need to be optimised. The missing data limited further interpretation of results; this needs to be considered in future studies. This study contributed to the establishment of NDRLs, regional DRLs and assisted DGMAH in optimising radiation dose. All centres involved in radiation imaging are urged to establish LDRLs to enhance the estimation of NDRLs for further optimisation.
